# Dasatinib anhydrate containing oral formulation improves variability and bioavailability in humans

**DOI:** 10.1038/s41375-023-02045-1

**Published:** 2023-10-03

**Authors:** Jiří Hofmann, Aleš Bartůněk, Tomáš Hauser, Gregor Sedmak, Josef Beránek, Pavel Ryšánek, Martin Šíma, Ondřej Slanař

**Affiliations:** 1 Zentiva, k.s., Prague, Czech Republic; 2 Institute of Pharmacology, First Faculty of Medicine, Charles University and General University Hospital in Prague, Prague, Czech Republic

**Keywords:** Drug development, Chronic myeloid leukaemia

## Abstract

Dasatinib monohydrate indicated for the treatment of chronic myeloid leukemia displays pH-dependent solubility. The aim of reported development program of novel dasatinib anhydrate containing formulation was to demonstrate improved absorption and lower pharmacokinetic variability compared to dasatinib monohydrate. In a bioavailability study comparing formulations containing 110.6 mg and 140 mg of dasatinib as anhydrate and monohydrate, respectively, both C_max_ and AUC of dasatinib were within standard 80.00–125.00% range, while the intra- and inter-subject variability for AUC_0-inf_ after the test product was approximately 3-fold and 1.5-fold less than after the reference, respectively.

In a drug–drug interaction study, omeprazole 40 mg reduced the mean AUC_0-inf_ of dasatinib by 19%, when the test was ingested 2 h before the 5th omeprazole dose. This decrease of exposure is clinically irrelevant and substantially less than after the reference. Co-prescription analysis supports the importance of pH-dependent solubility of dasatinib, as >21% of patients were treated concomitantly with a PPI and dasatinib despite warnings against this co-medication in the SmPC.

The novel dasatinib anhydrate containing formulation demonstrated improved absorption and less pharmacokinetic variability compared to dasatinib monohydrate product, which may translate into improved clinical outcomes, although this needs to be proven by an appropriate trial.

## Introduction

Dasatinib, a tyrosine kinase inhibitor, is primarily used in the treatment of chronic myeloid leukemia and Philadelphia chromosome-positive acute lymphoblastic leukemia. Oral administration of dasatinib is convenient and promotes improved quality of life for patients. However, compared to conventional intravenous treatment, oral use introduces risk of variable drug exposure, which may translate into decreased drug efficacy or variable safety.

Although fixed dosing is approved, later evidence and real life experience suggest that due to the high variability therapeutic drug monitoring should be implemented whenever possible [1].

An aqueous solubility is considered to account for significant variability in the drug absorption [2]. In-vitro data indicate a pH-dependent solubility of dasatinib monohydrate. The solubility decreases dramatically at pH values greater than 4.0, from 18.4 mg/mL at pH 2.6 to 0.205 mg/mL at pH 4.28 and only <0.001 mg/mL at pH 6.99 [3, 4].

Consequently, significant pharmacokinetic interactions have been also described between tyrosine kinase inhibitors and gastric pH-increasing drugs. Dasatinib co-administration with famotidine and antacids led to AUC decrease of ~60% and ~55%, respectively [4]. On the other hand, co-administration with betaine hydrochloride, a re-acidifying agent, prevented dasatinib AUC decrease induced by rabeprazole by 78% [5]. Therefore, H_2_ antagonists and proton pump inhibitors are not recommended for concomitant use with dasatinib monohydrate, while aluminum hydroxide/magnesium hydroxide products should be considered instead and administered up to 2 h prior to, or 2 h following the administration of dasatinib [6]. However, co-prescription analysis conducted exclusively for Zentiva by IQVIA Commercial GmbH & Co. OHG (Frankfurt am Main, Germany) showed that 623, 612 and 669 patients out of 2595, 2868 and 3258 patients treated with dasatinib in Germany in the periods 11/2016 to 10/2017, 11/2017 to 10/2018 and 11/2018 to 10/2019, respectively, were co-prescribed dasatinib and a PPI. It corresponds to 24%, 21% and 21% of all patients taking dasatinib in the respective time periods, who were co-prescribed dasatinib and a PPI in clinical practice, although this is explicitly discouraged in the reference product label.

Besides interacting drugs, reduced dasatinib absorption may also be expected under pathophysiological conditions that increase gastric pH. Reduced (hypochlorhydria) or absent (achlorhydria) production of gastric acid is common in the population and its prevalence increases with age [7].

There is a great potential for a common and under-recognized drug–drug interactions that could result in failure of therapy due to reduced exposure to key anticancer treatment [2, 8]. Time staggered dosing was proposed to mitigate potential impact of interaction between acid reducing agents and dasatinib to avoid the maximum effect of the acid reducing agent on drug absorption at the time of highest gastric pH. However, for PPIs a maximally staggered approach (dasatinib administered 22 h after the last dose of the PPI) still results in >40% reduction in dasatinib bioavailability [6, 9].

To mitigate the pH dependency and variability of exposure, dasatinib anhydrate containing formulation was developed. Due to the altered solubility characteristics of the anhydrate form, suprabioavailability was expected and subsequently proof-of-concept dose finding bioavailability study suggested dose reduction by 21% to achieve comparable exposure to the reference product. The aim of the reported confirmatory clinical development program of dasatinib as anhydrate was to demonstrate improved absorption characteristics and diminished pharmacokinetic variability of dasatinib compared with dasatinib monohydrate.

## Methods

### In-vitro dissolution

Intrinsic dissolution rates were measured on Sirius inForm (Sirius Analytical Inc, Beverly, USA) using a rotation disk. Dissolution of compressed tablets at 37 °C was monitored by in-situ UV fiber optic probe to detect the drug appearing in the medium. The dissolution media consisted of 100 mM acetate/phosphate buffer pH 4.5, and stirring of the solution was continuous at a constant rate of 100 rpm. Intrinsic dissolution rate was calculated from the slope of mass-time data.

Dissolution of dasatinib powders was conducted in 900 mL of hydrochloric acid (10 mM) pH 2.0, acetate buffer pH 4.5 (28 mM acetic acid / 22 mM sodium acetate) and phosphate buffer pH 6.8 (50 mM KH_2_PO_4_ / 22 mM NaOH) using standard USP II apparatus (Sotax AG, Aesch, Switzerland). Rotation speed was set to 75 rpm for 45 min and then increased to 150 rpm for 15 min. Accurately weighed powders were introduced directly into media. Samples were automatically withdrawn, filtered through 2.5 µm filter and analyzed by UV spectroscopy (Analytik Jena GmbH+Co. KG, Jena, Germany) at 280 nm in a 5 mm cuvette.

### Bioavailability and drug–drug interaction (DDI) studies

#### Study designs

Both studies have been approved by the institutional review board and State Institute for Drug Control, Prague, Czech Republic under EudraCT No. 2019-001928-35 and 2019-002892-33, respectively. Both studies were conducted at Quinta-Analytica s.r.o., Prague, Czech Republic, in compliance with principles of the Declaration of Helsinki, International Council for Harmonization (ICH), Good Clinical Practice, and applicable regulatory requirements. All subjects gave informed consent prior to study participation.

The bioavailability study was a single-dose, open-label, randomized, 2-sequence, 4-period, fully replicated cross-over study aimed to demonstrate bioequivalence of the test formulation Daruph film-coated tablet (Zentiva, k.s., Czech Republic) (T), containing 110.6 mg of dasatinib as anhydrate, with reference product Sprycel film-coated tablet (Bristol-Myers Squibb Pharma EEI) (R), containing 140 mg of dasatinib as monohydrate. Eligible participants were randomly assigned to a sequence TRTR or RTRT. Study design is illustrated in Supplementary Fig. 1. The manufacturing of T involved standard manufacturing processes of wet-granulation, compression and tablet coating. R product was purchased in Germany.

The DDI study was a single-sequence, 2-period, open-label study to investigate the effects of an oral proton pump inhibitor (PPI) omeprazole (Helicid 40 mg capsules, Zentiva, k.s., Czech Republic) administered QD for 5 days on the pharmacokinetics (PK) of T when ingested 2 h prior to the last omeprazole dose (‘staggered approach’). All subjects received T alone in the first period and T under pre-treatment with omeprazole in the second period. Study design is illustrated in Supplementary Fig. 1.

In both studies, a wash-out period of at least 4 days was kept between the treatment administrations (half-life of 3–5 h) [6]. Subjects fasted for at least 10 h prior to dosing and no food was provided until 4 h after the dose, after which a standardized breakfast was served. Lunch, snack and dinner were served at 6, 9 and 12 (bioavailability study) or 13 h (DDI study) post-dose. Water was allowed *ad libitum* until 1 h prior to drug administration and from 4 h after drug administration. 200 mL of water was provided with drug and then at 2 h following drug administration.

Blood samples were collected in K2EDTA containing tubes (BD Vacutainer, Plymouth, United Kingdom) at the following times: 0 (before dosing), 0.17, 0.33, 0.5, 0.67, 0.83, 1.0, 1.25, 1.5, 2.0, 2.5, 3.0, 3.5, 4.0, 5.0, 6.0, 8.0, 10.0, 12.0, 16.0, and 24.0 h after dosing. Following collection, samples were centrifuged at 2500 *g* for 8 min at 4 °C. Plasma obtained was separated, frozen on dry ice and stored at ≤ –20 °C until assayed.

#### Subjects

Healthy adult male and non-pregnant, non-breast-feeding female volunteers, aged 18 to 55 years with body mass index 18.5 and 30 kg/m^2^, non- or ex-smokers were eligible to participate in both studies. Eligibility was determined based on medical history, physical examination, ECG, clinical chemistry, urinalysis, hematology, viral serology, drug screen, alcohol breath test, urinary cotinine test and gastric pH measurements (only bioavailability study). Subjects were excluded if they had seated heart rate <50 or >100, seated blood pressure <90/60 or >140/90 mmHg, hypersensitivity to the study (co-)medication, positive HIV test, hepatitis B surface antigen or hepatitis C virus test, history of significant gastrointestinal, liver and kidney disease. Subjects with history of drug, tobacco, or alcohol abuse, or evidence of such abuse were excluded from studies. Other key exclusion criteria were acute or chronic disease, clinical findings that might have influenced the drug bioavailability, female subjects with positive pregnancy test, use of any prescription medicines <28 days before dosing, use of any over-the-counter medication and food supplements <14 days before dosing.

Additional screening was scheduled for subjects in the bioavailability study. Volunteers underwent gastric pH measurements using Digitrapper pH-Z and pH 400 recorder (Given Imaging Ltd., Yokneam, Israel) equipped with nasogastric catheter to confirm normochlorhydria defined as median gastric pH below 4 during a 4 h fasting measurement. Volunteers with median pH value above 4 were not allowed to enter the study.

#### Pharmacokinetic assessments

The plasma concentration-time profiles and pharmacokinetic parameters determined for dasatinib included maximum plasma concentration (C_max_), time to reach the maximum plasma concentration (T_max_), area under the plasma concentration-time curve from time zero to the time of the last quantifiable concentration (AUC_0-t_, measured by linear trapezoid method), AUC from time zero to infinity (AUC_0-inf_), terminal elimination half-life (t_half_). The pharmacokinetic parameters were determined by non-compartmental methods using Phoenix WinNonlin, version 8.1 (Certara, USA).

#### Statistical methods

Arithmetic mean and standard deviation were summarized for the main pharmacokinetic parameters. For T_max_, median and range were determined. In the bioavailability study, natural logarithm transformed PK parameters (C_max_, AUC_0-t_, AUC_0-inf_) were analyzed by a linear model with subject effect (nested within sequence), treatment, period and sequence as fixed effects. Fixed effects of subject and treatment were used in the analysis of ln-transformed PK parameters from the DDI study. The ln-transformed PK parameters for each of the product separately were analyzed by a linear model containing the terms for subject effect (nested within sequence), period and sequence for the estimation of intra-individual variability for T and R products. The intra-individual coefficient of variation (intra-CV) was estimated based on formula 100 × sqrt[exp(MSE)-1] [%], where MSE is the mean square error obtained from the ANOVA model of the ln-transformed parameters. Inter-subject coefficient of variation (inter-CV) was estimated from ln-transformed data based on formula 100 × sqrt[exp(s2)-1] [%], where s2 is the variance on the ln-scale. Two-sided 90% confidence intervals of the ratio of geometric least-square means derived from the exponential of the difference between the comparisons were calculated. In the bioavailability study, assessment of bioequivalence was based on the EU reference scaling method for C_max_ [10], while average bioequivalence with standard acceptance limits 80.00 to 125.00% was applied for AUC_0-t_. The assessment of PK-interaction was based on mean extent of absorption (the geometric mean ratio of AUC_0-t_ for dasatinib with PPI vs. dasatinib was to be 70.00 to 142.86% to exclude significant interaction).

Forty enrolled subjects into the bioavailability study were anticipated to lead to at least 36 completers, which would provide ≥80% probability that the 90% confidence interval for a geometric mean ratio for PK parameters would fit within 80.00% to 125.00%. The above calculation assumed that treatment differences were ≤5% and that C_max_ and AUC were log-normally distributed with an estimated intra-CV of 41%. In the DDI study, 36 enrolled subjects were expected to obtain at least 32 completers. This sample size was based on targeting an adequate precision for at least 20% difference in estimation of AUC_0-t_ between dasatinib administered in combination with omeprazole and dasatinib alone. The above calculation assumed an estimated intra-CV of about 47%. Statistical analysis was generated using SAS version 9.4 (SAS Institute Inc., Cary, NC, USA) by means of the general linear model procedure. Comparison of intra- and inter-subject variances was performed by F-test in R (4.3.0, R Foundation for Statistical Computing, 2023).

#### Analytical methods

Dasatinib in plasma was determined by a validated liquid chromatography-tandem mass spectroscopic method. In brief, samples were precipitated by a mixture of 100 mM solution of zinc sulfate, water and acetonitrile (1:3:4; v/v) (or, alternatively by 80% acetonitrile in water) in presence of internal standard (d_8_-dasatinib; TLC Pharmaceutical Standards, Canada). In both studies, a Thermo Fisher Scientific HPLC/MS/MS system equipped with 1250 Transcend pumps and a PAL HTS autosampler was employed. A different variant of analytical method was used in the bioavailability or DDI study, depending on technical equipment. In the bioavailability study, chromatographic separation was achieved with an isocratic elution on a Kinetex Phenyl Hexyl (5 mm, 50 × 3 mm) analytical column fitted with Luna C18(2) Mercury (5 mm, 20 × 4 mm) guard column from Phenomenex (USA). Mobile phase consisted of MeOH, 1% formic acid and water (50:15:35; v/v) at flow rate 0.5 mL/min. In the DDI study, samples were loaded onto a Kinetex Biphenyl 100 Å (5 mm, 50 × 3 mm) analytical column connected to Kinetex Biphenyl 100 Å (5 mm, 20 × 4 mm) guard column (Phenomenex, USA). Mobile phases consisted of MeOH, ammonium formate (20 mM) and water, a gradient elution at flow rate 0.5 mL/min was applied. Monitoring of the analyte and respective internal standard was achieved using TSQ Vantage or TSQ Quantiva tandem mass spectrometer (Thermo Fisher Scientific, USA) equipped with a heated electrospray ionization source (HESI) operating in the positive ionization mode. Quantitation was performed using selected reaction monitoring (SRM) for the following mass transitions: *m/z* 488 to 401 for dasatinib, *m/z* 496 to 406 for d_8_-dasatinib. Data acquisition and analysis was performed in Xcalibur and LCquan software, respectively. The bioanalytical range for dasatinib was 0.25–250.00 ng/mL. The concentrations were calculated using a linear regression model with weighted least squares (weight = 1/c and 1/c^2^, where c is the nominal concentration of the respective calibration sample). The bioanalytical method was validated in line with requirements of EMA Guideline [11]. At LLOQ, within-run accuracy was within the range of 90.42–116.22% and within-run precision was within the range of 3.72–4.33%. Similarly, between-run accuracy was 105.90% and between-run precision 11.50%. Within-run accuracy was within the range of 99.01–107.51% and within-run precision was within the range of 0.76–5.75% above LLOQ. Similarly, between-run accuracy was within the range of 101.75–104.82% and between-day precision was within the range of 1.75–4.68%. All validation parameters fulfilled the guideline acceptance criteria.

#### Safety assessments

Safety was evaluated through assessment of adverse events, clinical and laboratory test results, physical examination, and concomitant medication usage.

## Results

### In-vitro dissolutions

**Fig. 1:**
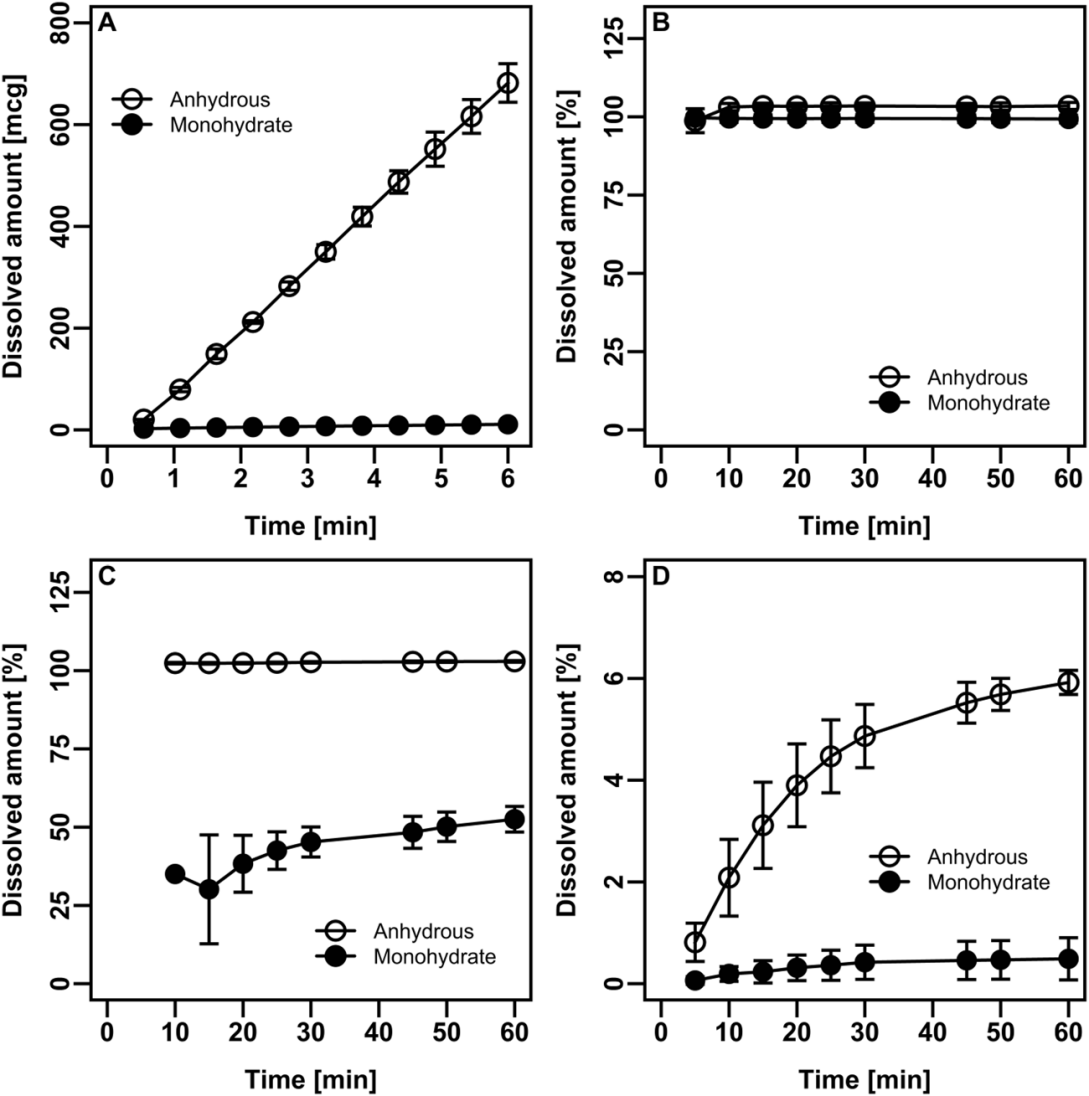
In vitro dissolutions. Intrinsic dissolution profiles of dasatinib polymorphs in acetate buffer pH 4.5 (**A**) and powder dissolution of dasatinib polymorphs in hydrochloric acid pH 2 (**B**), acetate buffer pH 4.5 (**C**), phosphate buffer pH 6.8 (**D**). Data are expressed as mean ± SD; *n* = 3.

The intrinsic dissolution of dasatinib drug substance in acetate buffer at pH 4.5 was significantly faster for the anhydrous polymorph compared to monohydrate form contained in the reference product (Fig. 1A). Anhydrous polymorph of dasatinib exhibited more than 80-fold higher intrinsic dissolution rate (1606 mcg/min/cm^2^) compared to monohydrate form (19 mcg/min/cm^2^). Dissolution of dasatinib powders in acidic media revealed that both forms of dasatinib have similar dissolution profiles (Fig. 1B), while difference was observed between the anhydrous and monohydrate form with increasing pH (Fig. 1C, D).

### Bioavailability study

#### Subject disposition and demography

Forty volunteers with normal gastric acid production were enrolled and completed the study. Demographic and baseline characteristics of the participants are summarized in Table 1.

**Table 1:** Demographic and other baseline characteristics.

	Bioavailability study	Drug–drug interaction study
Number of participants	40	36
Sex (males/females)	22/18	19/17
Age (years)^a^	39.6 ± 10.0	40.1 ± 9.1
Body weight (kg)^a^	76.7 ± 12.7	78.9 ± 10.8
Height (cm)^a^	174.2 ± 8.7	175.9 ± 8.0
Body mass index (kg/m^2^)^a^	25.1 ± 2.9	25.6 ± 3.0

^a^ Data are expressed as mean ± standard deviation.

#### Pharmacokinetic and bioavailability analysis

**Fig. 2:**
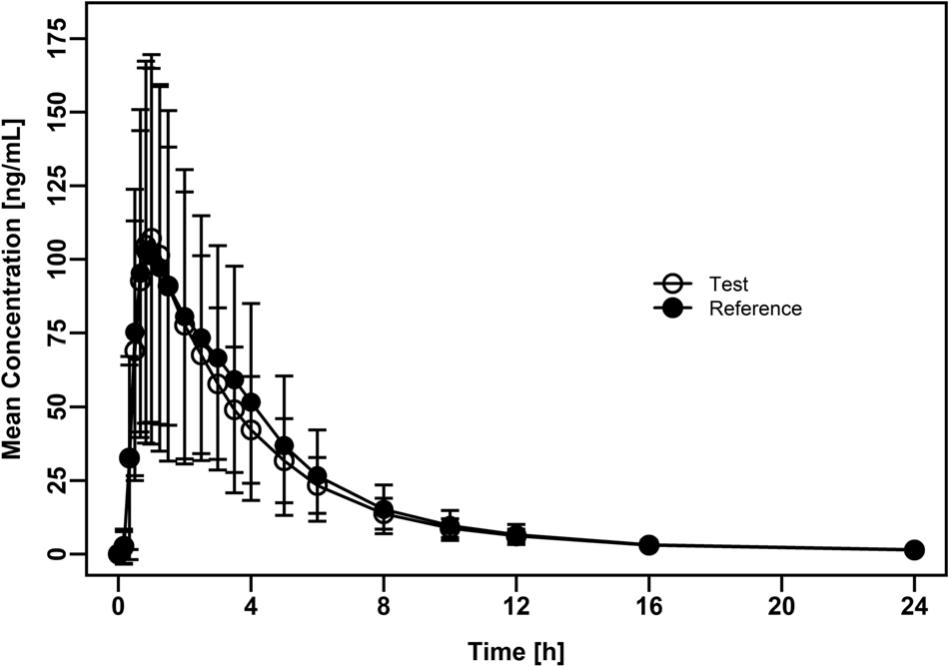
Bioavailability study. Arithmetic mean ( ± standard deviation) dasatinib plasma concentration-time profiles following single oral dose administration of test and reference formulations in healthy volunteers. Test: 110.6 mg of dasatinib anhydrate (*n* = 80); reference: 140 mg of dasatinib monohydrate (*n* = 80).

**Fig. 3:**
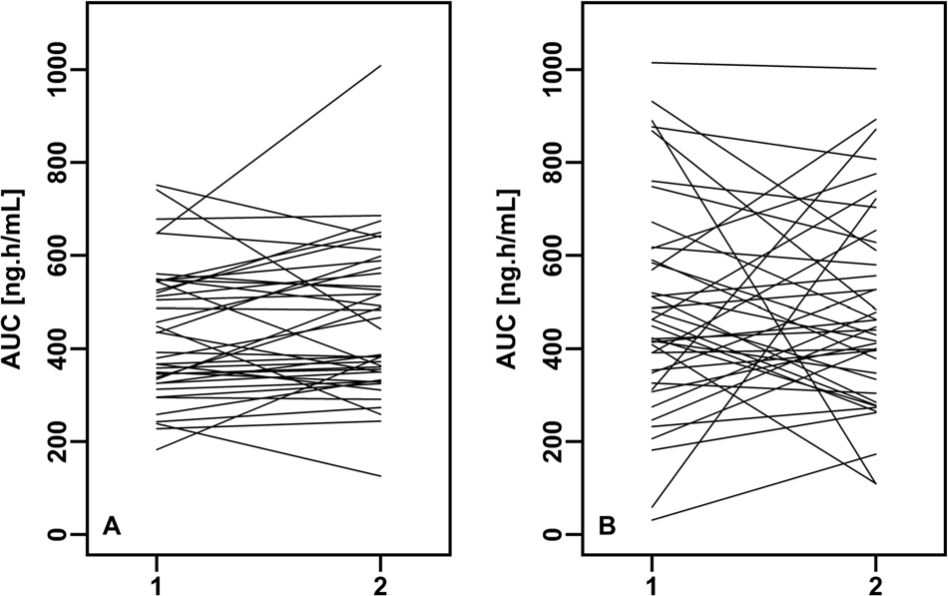
Variability in exposure after administration of reference and test formulation. Individual subject exposures of dasatinib under fasting conditions between the test (**A**) and the reference (**B**), plotted over corresponding study periods of bioavailability study. The lines connect the individual values of AUC_0-t_ for each subject between periods. 1, 2 – first, second administration of product.

The mean dasatinib plasma concentration-time profiles following oral administration of T and R formulation were almost identical (Fig. 2). For both treatments, the peak plasma concentrations of dasatinib were achieved at median of around 1 h, decreasing steadily afterwards with a mean estimated half-life of approximately 5 h. Total exposure of dasatinib was similar between both formulations, with mean ± SD AUC_0-inf_ values of 455.4 ± 154.3 and 499.5 ± 219.0 ng.h/mL for test and reference formulation, respectively. Mean C_max_ and AUC_0-t_ were also similar between formulations; the corresponding summary statistics of dasatinib PK parameters are provided in Table 2. The PK data from all subjects and all periods were included in the bioequivalence assessment. The geometric mean ratios and associated 90%CI for C_max_, AUC_0-t_ and AUC_0-inf_ were within 80.00 to 125.00% range (Table 2), demonstrating that the rate and extent of absorption of dasatinib is equivalent between the compared formulations. However, the variability in exposure was substantially lower for T formulation containing dasatinib anhydrate versus the monohydrate form present in the R, as visualized in the individual subjects’ AUC data plotted over corresponding study periods (Fig. 3). These data suggest that exposure of dasatinib after the administration of T (Fig. 3A) is more consistent when compared to the R (Fig. 3B). This conclusion is further supported numerically, by the calculated variability for the main plasma PK parameters. The intra-subject variability (expressed as coefficient of variation (CV%)) for AUC_0-t_ and AUC_0-inf_ for the T was approximately 3-fold lower than for the R (Table 3). For C_max_, the intra-subject variability was approximately 2.5-fold lower after the administration of T compared to the R (Table 3). The inter-individual variability was 1.6-fold, 1.8-fold and 1.5-fold lower for the T when compared to the R for C_max_, AUC_0-t_ and AUC_0-inf_ (Table 3), respectively.

**Table 2:** Pharmacokinetic parameters and geometric least-square means ratios (90% CI) of dasatinib following single-dose of test and reference formulations (A-bioequivalence study) and following oral dosing of test formulation with and without 40 mg omeprazole (PPI) administered in a staggered manner (B-drug–drug interaction study).

(A) Bioequivalence study			
PK-metric	Mean ± SD		GMR (90% CI)
	Test (*n* = 80)	Reference (*n* = 80)	Test vs. Reference
AUC_0-t_ [ng.h/mL]	444.66 ± 153.01	482.41 ± 221.40	99.36 (89.39–110.43)
AUC_0-inf_ [ng.h/mL]	455.41 ± 154.26	499.45 ± 218.99^b^	96.26 (88.05–105.23)
C_max_ [ng/mL]	132.29 ± 61.65	139.65 ± 66.64	100.39 (86.83–116.06)
t_max_^a^ [h]	1.00 (0.50–4.00)	0.83 (0.33–24.00)	–
t_half_ [h]	5.13 ± 1.31	5.00 ± 1.35^b^	–
**(B) Drug–drug interaction study**			
PK-metric	Mean ± SD		GMR (90% CI)
	Test+PPI (*n* = 35)	Test (*n* = 35)	Test+PPI vs. Test
AUC_0-t_ [ng.h/mL]	316.84 ± 117.47	398.95 ± 137.36	79.70 (72.56–87.54)
AUC_0-inf_ [ng.h/mL]	330.71 ± 117.62	408.96 ± 136.42	81.00 (74.63–87.93)
C_max_ [ng/mL]	81.87 ± 41.20	132.05 ± 60.49	62.34 (51.25–75.82)
t_max_^a^ [h]	1.25 (0.50–5.00)	0.83 (0.33–4.00)	–
t_half_ [h]	6.09 ± 2.07	5.44 ± 1.87	–

*CI* confidence intervals, *GMR* geometric mean ratio, *SD* standard deviation.

^a^median (range).

^b^*n* = 79.

**Table 3:** Intra- and inter-subject PK-variability of test (110.6 mg of dasatinib anhydrate) and reference formulation (140 mg of dasatinib monohydrate).

PK-metric	Intra-CV [%]^a^		Inter-CV [%]^b^	
	Test	Reference	Test	Reference
C_max_	31.0^†^	76.8	50.7^†^	83.2
AUC_0-t_	17.7^†^	56.1	36.7^†^	65.0
AUC_0-inf_	16.6^†^	48.6	35.5^*^	52.6

*P < 0.01; ^†^*P* < 0.001.

^a^based on residual mean square error from ln-transformed data for test and reference.

^b^based on ln-transformed data, corresponds to geometric CV.

#### Safety and tolerability

A total of 19 subjects (48%) experienced 44 drug related adverse events (AEs), of which 17 (42.5%) were related to administration of T and 27 (67.5%) to the R. The reported AEs and their CTC grading are shown in Table 4, all AEs were resolved at the end of the study.

**Table 4:** Reported adverse effects and their severity graded according to Common Terminology Criteria (CTC) in bioequivalence (A) and drug–drug interaction (B) study.

(A) Bioequivalence study					
Adverse effect	After Test		After Reference		Total
	Grade 1	Grade 2	Grade 1	Grade 2	
Fever	0	0	1	3	4
Diarrhea	0	0	0	2	2
Nausea	2	0	2	0	4
Vomiting	0	0	1	0	1
Headache	0	15	3	15	33
**(B) Drug–drug interaction study**					
Adverse effect	After dasatinib		After dasatinib+omeprazole		Total
	Grade 1	Grade 2	Grade 1	Grade 2	
Fever	0	1	0	0	1
Chills	1	0	0	0	1
Weakness	2	0	0	0	2
Nausea	3	1	0	1	5
Vomiting	1	0	0	0	1
Headache	1	6	0	4	11
Palmar exanthema	0	1	0	0	1
Neck exanthema	0	1	0	0	1

### Drug–drug interaction study

#### Subject disposition and demography

Totally 36 volunteers were enrolled to receive T containing 110.6 mg of dasatinib anhydrate either alone or in presence of omeprazole (40 mg q.d. for 5 days). One subject discontinued prior to second study period due to an AE (dermatitis). Demographic and baseline characteristics of the participants are summarized in Table 1.

#### Pharmacokinetic and bioavailability analysis

**Fig. 4:**
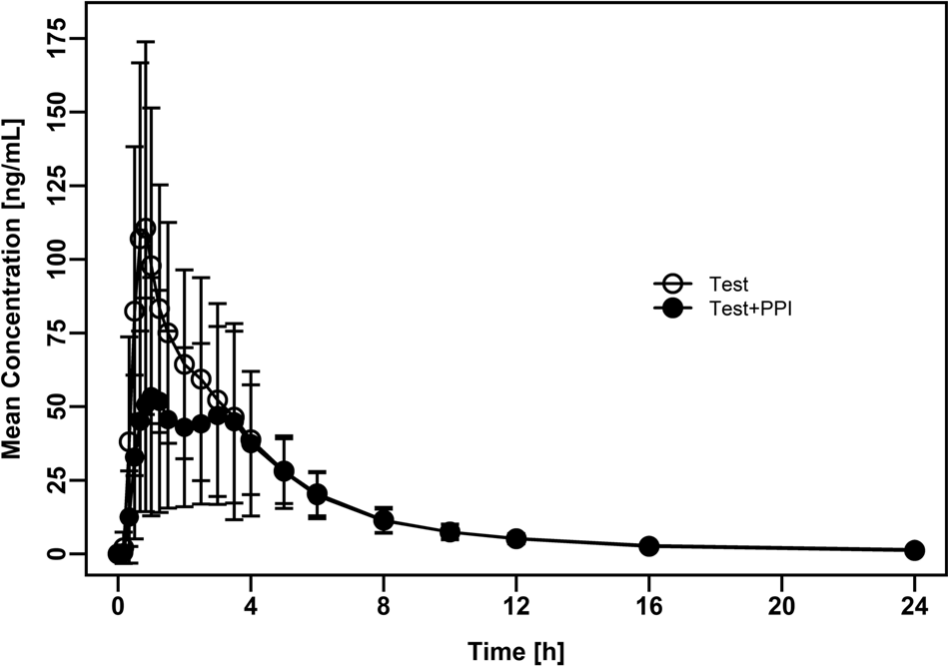
Drug-drug interaction study. Arithmetic mean ( ± standard deviation) dasatinib plasma concentration-time profiles following oral administration of test formulation (110.6 mg of dasatinib anhydrate) alone (Test) and with pre-treatment by omeprazole 40 mg, q.d. (Test+PPI) in healthy volunteers (*n* = 35).

The observed plasma concentration versus time profiles and summary statistics of dasatinib PK parameters following administration of dasatinib anhydrate in presence of omeprazole or alone are presented in Fig. 4 and Table 2. Median T_max_ of 1.25 h was observed following the co-administration of dasatinib anhydrate with omeprazole and 0.83 h following the administration of dasatinib anhydrate alone. As expected, pre-treatment with omeprazole prior to dasatinib reduced dasatinib exposure. The mean AUC_0-t_, AUC_0-inf_ and C_max_ decreased by 20%, 19% and 38%, respectively, when T was administered in a staggered manner, i.e., 2 h prior to the last dose of omeprazole. Individual PK profiles from the DDI study were plotted for test and reference and illustrated in the Supplementary Fig. 2.

#### Safety and tolerability

Total of 13 subjects (36%) experienced 23 treatment related AEs. The reported AEs and their CTC grading are shown in Table 4, all AEs experienced during the study were resolved at the end of the study.

## Discussion

Previous evidence shows that the drug oral bioavailability does not only depend on the aqueous solubility as may be demonstrated for low solubility BCS II/IV compounds as naproxen, diazepam or phenytoin, which possess high absolute bioavailability >90% [12, 13]. Furthermore, elaboration of drug formulations to improve kinetics of solubility also does not always enhance low oral bioavailability [14]. Nevertheless, in our case, the >80-fold difference in aqueous solubility of the developed anhydrous form of dasatinib compared with monohydrate form at pH of 4.5 translated in approximately 20% increase of bioavailability in normogastric population and about 3.5-fold higher bioavailability in subjects with gastric pH >4 (data on file).

Overall, the increased bioavailability allowed a 21% reduction of the administered dose for T containing dasatinib anhydrate compared to R containing dasatinib monohydrate. This does not affect the efficacy/safety, as the rate and extent of exposure in the bioequivalence study were equivalent. However, the 21% reduction in the amount of dasatinib reaching the market is a significant environmental benefit. Considering a daily dose of 100 mg taken by 3258 patients in Germany in 2019, the annual consumption in Germany was approximately 119 kg of dasatinib. If the 447 million EU population were treated in a similar way, the annual consumption in the EU countries would be approximately 640 kg. A 21% reduction in the therapeutic dose would mean a reduction by 134 kg of dasatinib per year in the EU environmental burden.

A substantially reduced pH dependence of exposure after administration of dasatinib anhydrate (T) was observed when the drug was co-administered with omeprazole. In this co-medication, the average exposure to dasatinib was reduced by only approximately 20%. This magnitude of exposure reduction is likely insignificant because it is similar to other interactions with dasatinib that do not lead to contraindications or dosage adjustments, e.g. concomitant use of dexamethasone. According to the label, co-administration of dexamethasone decreases AUC of approximately 25%, which is unlikely to be clinically significant [6]. The DDI study design included administration of dasatinib 22 h after omeprazole dose, i.e. 2 h prior the last omeprazole dose. This approach was previously proposed as appropriate to manage significant interaction between TKIs and PPIs [15]. Also, it allows comparison with the clinical development program of the original product, whose DDI study included identical time interval between dasatinib and a PPI. Under identical PPI administration timing, mean exposure of the reference formulation expressed as AUC and C_max_ was reduced substantially more, i.e. by 43% and 42%, respectively, according to the reference product label [6]. These data indicate that the oral formulation containing dasatinib anhydrate displays lower absorption interaction in presence of acid modifiers such as PPI. Although PPIs are known irreversible covalent inhibitors of proton pump, the gastric acid secretion is not consistently blocked over the 24 h dosing interval [16, 17]. Intragastric pH is expected to start to decrease within 12-14 h after PPI administration [15], and therefore the time window used between PPIs and dasatinib in the DDI study does not describe possible worst-case interaction scenario, but reflects a clinically used approach to minimize the impact of reduction of exposure to dasatinib [6, 15].

An important outcome of the development program with potential clinical relevance is the reduction in the pharmacokinetic variability of dasatinib after administration of T in the form of dasatinib anhydrate compared to R. While the intra-subject variability of T was reduced to approximately one-third of the variability observed after R, the inter-subject variability was reduced to approximately 56% of that of R. The substantial reduction in the variability of dasatinib plasma levels and exposure may translate into clinical outcomes as for predictability of therapeutic response, low intra-individual variability is an essential factor to set the optimal dosage for an individual patient. Especially for the drugs that do not possess wide therapeutic index, low intra-subject variability of PK and PK/PD are the key characteristics [18].

Dasatinib exhibits exposure time-dependent effect where plasma concentrations above inhibitory concentration (IC_50_ CD_34+_ cells) for more than 12.8 h led to a better clinical response [19]. Therefore, the efficacious levels may be expected to be a prerequisite for sufficient therapeutic response and well predictable in case of low variation. It has been repeatedly reported that dasatinib exposure possesses high variability [20], and attempts to individualize drug dosing either using TDM or other measures have not been widely implemented due to insufficiently defined metrics and target exposure. Therefore, 3-fold reduction of intra-subject variability after novel dasatinib anhydrous formulation compared with the reference formulation may provide an important step towards improved treatment response predictability of dasatinib. There are no appropriately designed randomized clinical trials to directly confirm that the high exposures or low PK-variability of dasatinib translate into clinical treatment outcomes, nevertheless associations have been observed in retrospective analyses that shown decreased progression free survival and overall survival in patients treated with gastric pH-increasing drugs [21, 22]. In a cohort of 12,538 patients treated with TKIs, co-administration of PPIs (22.7% patients) was associated with increased risk of death in 90 days (hazard ratio 1.16) and in one year (hazard ratio 1.10) [21]. In retrospective analysis from the Swedish CML registry, the estimated 5-year survival was lower for TKI-treated CML patients with vs without PPI (79% vs. 94%). This corresponds to a significantly increased crude hazard ratio of death of 3.5 (95%CI, 2.1–5.3, *p* < 0.0001) [23]. Finally, co-administration of PPI or H_2_-receptor antagonists was associated with shorter median progression free survival (1.4 vs. 2.3 months, *p* < 0.001) and shorter median overall survival (12.9 vs. 16.8 months, *p* = 0.003) in patients treated with erlotinib for non-small-cell lung carcinoma [22].

Despite warnings, co-medication with PPI is very common among dasatinib-treated CML patients in a real-world setting. The importance of this extrinsic source of pH-dependent failure in dasatinib exposure in the form of monohydrate is diminished when the drug is administered in the form of novel anhydrous formulation. Similarly, intrinsic factors leading to hypochloremia will have only reduced impact on PK performance of the newly developed product. The reduced pharmacokinetic variability of dasatinib in the population after oral administration of novel anhydrous formulation is likely to translate into improved clinical outcomes, although this needs to be proven by an appropriately designed clinical trial.

### Supplementary information

Supplementary data

## Data Availability

The data that support the findings of this study are available from the corresponding author upon reasonable request.

## References

[1] Rousselot P, Mollica L, Guilhot J, Guerci A, Nicolini FE, Etienne G (2021). Dasatinib dose optimisation based on therapeutic drug monitoring reduces pleural effusion rates in chronic myeloid leukaemia patients. Br J Haematol.

[2] Budha NR, Frymoyer A, Smelick GS, Jin JY, Yago MR, Dresser MJ (2012). Drug absorption interactions between oral targeted anticancer agents and PPIs: is pH-dependent solubility the Achilles heel of targeted therapy?. Clin Pharm Ther.

[3] European Medicines Agency. Sprycel: European public assessment report. *First published*: 18/08/2009. Last updated: 29/03/2019. https://www.ema.europa.eu/en/documents/overview/sprycel-epar-medicine-overview_en.pdf.

[4] Eley T, Luo FR, Agrawal S, Sanil A, Manning J, Li T (2009). Phase I study of the effect of gastric acid pH modulators on the bioavailability of oral dasatinib in healthy subjects. J Clin Pharm.

[5] Yago MR, Frymoyer A, Benet LZ, Smelick GS, Frassetto LA, Ding X (2014). The use of betaine HCl to enhance dasatinib absorption in healthy volunteers with rabeprazole-induced hypochlorhydria. AAPS J.

[6] European Medicines Agency. Sprycel: Summary of product characteristics. *First authorisation*: 20/11/2006. Latest renewal: 15/7/2016. https://www.ema.europa.eu/en/documents/product-information/sprycel-epar-product-information_en.pdf.

[7] Christiansen PM (1968). The incidence of achlorhydria and hypochlorhydria in healthy subjects and patients with gastrointestinal diseases. Scand J Gastroenterol.

[8] Smelick GS, Heffron TP, Chu L, Dean B, West DA, Duvall SL (2013). Prevalence of acid-reducing agents (ARA) in cancer populations and ARA drug-drug interaction potential for molecular targeted agents in clinical development. Mol Pharm.

[9] Zhang L, Wu F, Lee SC, Zhao H, Zhang L (2014). pH-dependent drug-drug interactions for weak base drugs: potential implications for new drug development. Clin Pharmacol Ther.

[10] European Medicines Agency. Guideline on the investigation of bioequivalence. CPMP/EWP/QWP/1401/98 Rev. 1/Corr**. 2010. https://www.ema.europa.eu/en/documents/scientific-guideline/guideline-investigation-bioequivalence-rev1_en.pdf.

[11] European Medicines Agency. Guideline on bioanalytical method validation. EMEA/CHMP/EWP/192217/2009 Rev. 1 Corr. 2**. 2011. https://www.ema.europa.eu/en/documents/scientific-guideline/guideline-bioanalytical-method-validation_en.pdf.

[12] Yazdanian M, Briggs K, Jankovsky C, Hawi A (2004). The “high solubility” definition of the current FDA guidance on biopharmaceutical classification system may be too strict for acidic drugs. Pharm Res.

[13] Faassen F, Vromans H (2004). Biowaivers for oral immediate-release products: implications of linear pharmacokinetics. Clin Pharmacokinet.

[14] Kuentz M, Nick S, Parrott N, Rothlisberger D (2006). A strategy for preclinical formulation development using GastroPlus as pharmacokinetic simulation tool and a statistical screening design applied to a dog study. Eur J Pharm Sci.

[15] van Leeuwen RWF, Jansman FGA, Hunfeld NG, Peric R, Reyners AKL, Imholz ALT (2017). Tyrosine kinase inhibitors and proton pump inhibitors: an evaluation of treatment options. Clin Pharmacokinet.

[16] Hunfeld NG, Touw DJ, Mathot RA, van Schaik RH, Kuipers EJ (2012). A comparison of the acid-inhibitory effects of esomeprazole and rabeprazole in relation to pharmacokinetics and CYP2C19 polymorphism. Aliment Pharm Ther.

[17] Blum RA, Hunt RH, Kidd SL, Shi H, Jennings DE, Greski-Rose PA (1998). Dose-response relationship of lansoprazole to gastric acid antisecretory effects. Aliment Pharmacol Ther.

[18] Habet S (2021). Narrow therapeutic index drugs: clinical pharmacology perspective. J Pharm Pharmacol.

[19] Ishida Y, Murai K, Yamaguchi K, Miyagishima T, Shindo M, Ogawa K (2016). Pharmacokinetics and pharmacodynamics of dasatinib in the chronic phase of newly diagnosed chronic myeloid leukemia. Eur J Clin Pharmacol.

[20] He S, Bian J, Shao Q, Zhang Y, Hao X, Luo X (2021). Therapeutic drug monitoring and individualized medicine of dasatinib: focus on clinical pharmacokinetics and pharmacodynamics. Front Pharmacol.

[21] Sharma M, Holmes HM, Mehta HB, Chen H, Aparasu RR, Shih YT (2019). The concomitant use of tyrosine kinase inhibitors and proton pump inhibitors: prevalence, predictors, and impact on survival and discontinuation of therapy in older adults with cancer. Cancer.

[22] Chu MP, Ghosh S, Chambers CR, Basappa N, Butts CA, Chu Q (2015). Gastric acid suppression is associated with decreased erlotinib efficacy in non-small-cell lung cancer. Clin Lung Cancer.

[23] Larfors G, Lennernäs H, Liljebris C, Brisander M, Jesson G, Andersson P (2022). Comedication of proton pump inhibitors and dasatinib is common in CML but XS004, a novel amorphous solid dispersion formulation of dasatinib, provides improved uptake and low pH-dependency, minimizing unwanted drug-drug interactions. Blood.

