# Supplementary material for: Dasatinib anhydrate containing oral formulation improves variability and bioavailability in humans
Source: Leukemia. 2023 Oct 3;37(12):2486–92. doi: 10.1038/s41375-023-02045-1 (PMC10681895; doi:10.1038/s41375-023-02045-1)

Supplementary figure 1. Studies design flowchart.

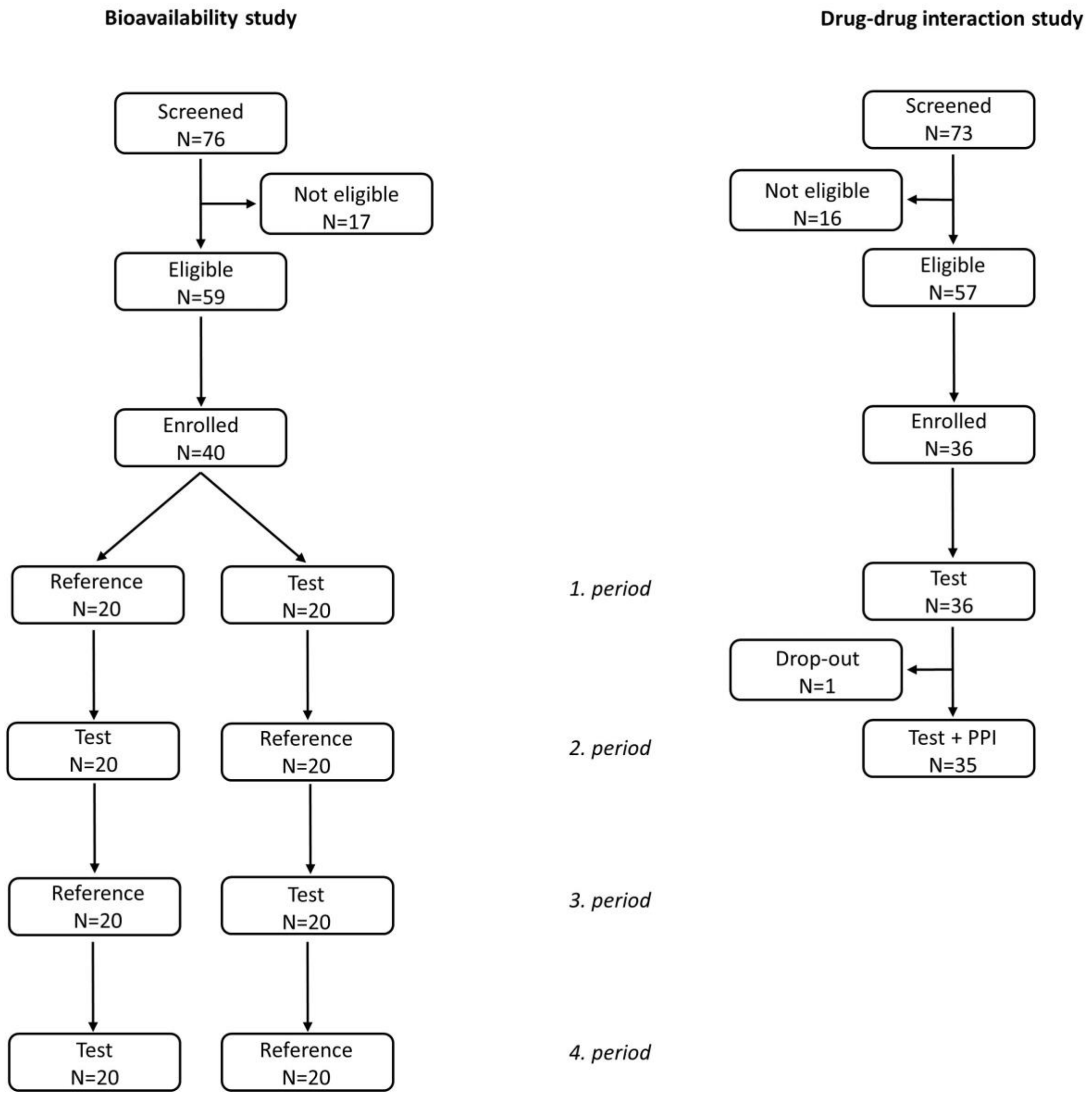

Supplementary figure 2. Individual plasma concentration-time profiles of dasatinib following oral administration of test formulation (110.6 mg of dasatinib anhydrate) alone (A) and with pre-treatment by omeprazole 40 mg, q.d. (B) in healthy volunteers.

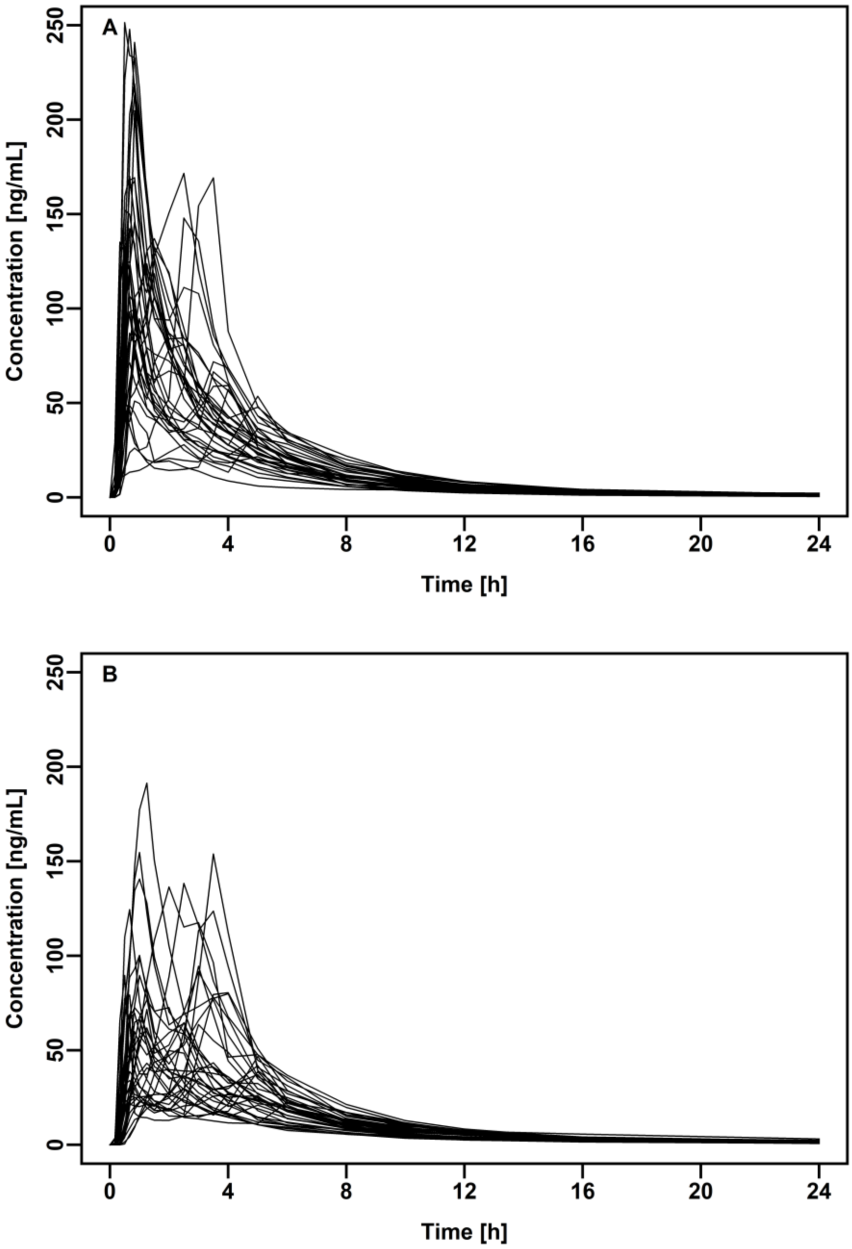

Supplement: Supplementary file 1 — Supplementary data [file 41375_2023_2045_MOESM1_ESM.pdf]
